# Predicting 3D Structure, Cross Talks, and Prognostic Significance of *KLF9* in Cervical Cancer

**DOI:** 10.3389/fonc.2021.797007

**Published:** 2022-01-03

**Authors:** Sadia Safi, Yasmin Badshah, Maria Shabbir, Kainat Zahra, Khushbukhat Khan, Erum Dilshad, Tayyaba Afsar, Ali Almajwal, Nawaf W. Alruwaili, Dara Al-disi, Mahmoud Abulmeaty, Suhail Razak

**Affiliations:** ^1^ Department of Healthcare Biotechnology, Atta-ur-Rahman School of Applied Biosciences, National University of Sciences and Technology, Islamabad, Pakistan; ^2^ Department of Bioinformatics and Biosciences, Faculty of Health and Life Sciences, Capital University of Science and Technology (CUST), Islamabad, Pakistan; ^3^ Department of Community Health Sciences, College of Applied Medical Sciences, King Saud University, Riyadh, Saudi Arabia

**Keywords:** cervical cancer, microRNA 223, *PKCϵ*, PI3K/Akt signaling pathway, Ramachandran plots, *KLF9*

## Abstract

Our study aimed to identify the new blood-based biomarkers for the diagnosis and prognosis of cervical cancer. Moreover, the three-dimensional (3D) structure of Kruppel-like factor 9 (*KLF9*) was also determined in order to better understand its function, and a signaling pathway was constructed to identity its upstream and downstream targets. In the current study, the co-expressions of tumor protein D52 (*TPD52*), *KLF9*, microRNA 223 (miR-223), and protein kinase C epsilon (*PKCϵ*) were evaluated in cervical cancer patients and a possible relation with disease outcome was revealed. The expressions of *TPD52*, *KLF9*, miR-223, and *PKCϵ* were studied in the blood of 100 cervical cancer patients and 100 healthy controls using real-time PCR. The 3D structure of *KLF9* was determined through homology modeling *via* the SWISS-MODEL and assessed using the Ramachandran plot. The predicted 3D structure of *KLF9* had a similarity index of 62% with its template (*KLF4*) with no bad bonds in it. In order to construct a genetic pathway, depicting the crosstalk between understudied genes, STRING analysis, Kyoto Encyclopedia of Genes and Genomes (KEGG), and DAVID software were used. The constructed genetic pathway showed that all the understudied genes are linked to each other and involved in the PI3K/Akt signaling pathway. There was a 23-fold increase in *TPD52* expression, a 2-fold increase in miR-223 expression, a 0.14-fold decrease in *KLF9* expression, and a 0.05-fold decrease of *PKCϵ* expression in cervical cancer. In the present study, we observed an association of the expressions of *TPD52*, *KLF9*, miR-223, and *PKCϵ* with tumor stage, metastasis, and treatment status of cervical cancer patients. Elevated expressions of *TPD52* and miR-223 and reduced expressions of *KLF9* and *PKCϵ* in peripheral blood of cervical cancer patients may serve as predictors of disease diagnosis and prognosis. Nevertheless, further *in vitro* and tissue-level studies are required to strengthen their role as potential diagnostic and prognostic biomarkers.

## Introduction

Cervical cancer arises from the cervix in women. It is the fourth most prevalent and the fourth most frequent cause of cancer mortality, with approximately 604,000 new cases and 342,000 causalities all over the world in 2020 (1). Various studies have confirmed the association between genital human papillomavirus (HPV) and cervical cancer. Sexual contact is the key risk factor associated with HPV acquisition. HPV has been recommended as solely the “necessary cause” of cervical cancer (2). Pap smear has been the most widely used cervical cytology screening technique for the past 50 years. However, the Pap smear is far from perfect, and its foremost shortcoming is the possibility of a false-negative result (3). No significant improvements in the Pap test have been made, due to which false-negative results that arise from the Pap test are continuously being reported even now. Laboratory misinterpretations, preparation errors, and improper sampling are the main causes of erroneous negative results (4). Although the basic treatment for cervical cancer is surgery or chemoradiation therapy, patients with advanced-stage tumor have poor disease prognosis with severe side effects. Hence, substitute screening approaches are required in underdeveloped and developing countries (5).

It has been reported that *KRAS* and phosphoinositide 3-kinases, upon activation *via* different receptors, e.g., G protein-coupled receptors (GPCRs) and receptor tyrosine kinase (RTK), cause the activation of the major downstream signaling pathways. Various studies have confirmed the interactions of Kruppel-like factor 9 (*KLF9*), protein kinase C epsilon (*PKCϵ*), tumor protein D52 (*TPD52*), and microRNA 223 (miR-223) with the downstream components of these signaling pathways, which eventually lead to carcinogenesis (6–9).

*TPD52* (CR542034.1) is situated at the 8q21 chromosome, on an area that is commonly amplified in numerous cancers particularly in humans (10). The primary evidence of the importance of an altered expression of *TPD52* in various cancers was obtained from the position of this gene on chromosome 8q, and during the mid-1990s, it became widely understood that the expression of *TPD52* increases in certain tumor types, as well as in *MYC* oncogene. Nevertheless, the role of *TPD52* in the onset of cancer is still debatable (11). The expression of *TPD52* is upregulated in certain types of cancers, such as breast, prostate, ovarian, and pancreatic cancer, Burkitt’s lymphoma, multiple myeloma, and melanoma (12). On the other hand, the expression of *TPD52* is also downregulated in other cancer types such as leiomyosarcoma, papillary renal cell cancer, clear cell renal cell cancer, lung cancer, and liposarcoma. Due to its altered expression in various cancers, it is referred to as a controversial gene (13). Several studies have reported evidence of the role of *TPD52* in various signaling pathways of cancers, i.e., in the PI3K/Akt signaling pathway (14), protein kinase B/Akt signaling pathway (15), and nuclear factor-κB transactivation (16).

*KLF9* (NM_001206.4) is a regulator of transcription in cellular adhesion, differentiation, and proliferation in the endometrium (17). Irregular expression of *KLF9* may contribute toward the onset of several carcinomas and their proliferation (18). *KLF9* is known to interact substantially with the Akt pathway. One of the studies validated the involvement of *KLF9* in the Akt pathway and indicated that *KLF9* substantially inhibits AKT activation and abrogates tumor growth in prostate cancer (19, 20).

PKCϵ (NM_005400.3) is one of the members of the protein kinase C family. Out of 10 isoforms of serine/threonine kinases, PKCϵ is the most widely studied for its contribution to malignant transformation (21). A recent study has revealed the interaction of PKCϵ with Akt, suggesting that the downregulation of *PKCϵ* causes the inhibition of Akt in breast cancer cells, thus increasing drug efficacy in breast cancer patients (22). The overexpression of *PKCϵ* has been reported in a wide range of carcinomas, including breast cancer, lung cancer, prostate cancer (23, 24), and brain tumors (25).

Similarly, recent studies have suggested the reduced expression of miR-223 (NC_000023.11) in metastatic and end-stage osteosarcoma patients, indicating the inhibitory role of miR-223 in osteosarcoma. An increased expression of miR-223 revokes atherosclerosis advancement by activating the PI3K/AKT pathway through blockade of *TLR4* signaling. Its dysregulation is also associated with aberrant Akt/mTor pathway in various diseases such as myocardial infarction (26), colorectal cancer (27), and pancreatic cancer (28).

Kruppel-like factor (KLF) proteins have been found in diverse species and are known to have evolved by gene duplication (29, 30). However, the structures of all KLFs, except that of *KLF4* (PDB ID: 2BWU), remain unpredicted. The prediction of the first ever structure of *KLF4* provided new insights toward a better understanding of the molecular basis and functional anatomy of *KLF4* and the other members of the KLF family (31) The three-dimensional (3D) structure of proteins helps in understanding their functions and their interactions with their binding partners (32). Our study describes the approaches to identify and determine the conserved domains and 3D structure of *KLF9* and the development of a genetic pathway, thus establishing a crosstalk between *KLF9* and its upstream and downstream targets. Additionally, although the individual expression status of *TPD52*, *KLF9*, miR-223, and *PKCϵ* has been previously studied in various tumors, no study has investigated the co-expressions of *TPD52*, *KLF9*, miR-223, and *PKCϵ* in any cancer type. Hence, our study also aimed to identify the combined expression patterns of *TPD52*, *KLF9*, miR-223, and *PKCϵ*, and their relationship with clinicopathological features, and to investigate the diagnostic and prognostic value of these genes in cervical cancer patients.

## Methods

### Blood Sample Collection

Blood samples were collected only from those patients who gave approval to collect their blood voluntarily in Combined Military Hospital (CMH), Rawalpindi, after approval by the Ethical Committee of Combined Military Hospital and ASAB, National University of Science and Technology, Islamabad, Pakistan. All participants were informed about the study objectives and signed the informed consent. The study protocol was carried out in accordance with the principles of the Declaration of Helsinki (33).

Blood samples were collected from female patients with histologically confirmed diagnosis of localized and/or metastasized carcinoma of the cervix (*n* = 100) and currently were on chemotherapy, radiotherapy, or chemoradiotherapy. Patients with co-infection of HIV were excluded from our study. The median age of cervical cancer patients was 47.5 years (range, 35–60 years). Furthermore, a control group was also included in the present study, which comprised blood samples from healthy individuals (*n* = 100), for accurate interpretion of the results.

### RNA Extraction and cDNA Synthesis

RNA was extracted from whole blood drawn from peripheral veins of cancer patients using the TriZol reagent (Thermo Fischer Scientific, Waltham, MA, USA). The reaction was conducted on ice to avoid RNA degradation. For cDNA synthesis, 20 μl of the reaction mixture was prepared by adding 1 μl of Oligo dT20 [Random Hexamer, 1 μl dNTP mix (2.5 mM)], <5 μg of RNA, and RNAse-free water up to 10 μl. The reaction mixture was incubated at 65°C in a thermocycler for 5 min. In the next step, 10× reaction buffer (2 μl), 100 mM DTT (1 μl), RNase inhibitor (0.5 μl), and RTase (1 μl) were added into the PCR tube (same) and placed in a thermocycler for 50 min at 42°C and for 10 min at 70°C. The synthesized cDNA was stored at −20°C.

### Real-Time PCR

For analysis of the expression of the candidate gene and microRNA (miRNA), real-time PCR was used. Real-time reaction mixture was made by adding 10 μl of Wiz pure qPCR master mix (SYBR), 6 mM of forward and reverse primers, and 10 μg of cDNA with RNAse-free water up to a volume of 20 μl. The conditions for quantitative PCR (qPCR) amplification were 40 cycles with an initial temperature of 95°C for 10 min, which basically activated Hot Start DNA polymerase, followed by 95°C for 15 s and then amplification for 1 min for 61°C, followed by real-time analysis for 45 s at 75°C. The primer sequences and the GC (guanine–cytosine) content are presented in **Table 1**. The specificity of primers was confirmed by observing the melt curve analysis of qPCR. The reagent and software used for real-time PCR were SYBR Green dye and 7300 SDS software, respectively. For quantifying the gene expression, the 2^−ΔΔCT^ method was performed. Moreover, the Livak method was used for conversion of the cycle threshold (*C*_t_) values, obtained for real-time PCR, into fold change. β-actin was used as a control, and the experiment was performed in triplicate. The *C*_t_ values obtained in triplicate for each sample was found to be almost the same, hence confirming the validity of the results.

**Table 1:** Sequences and parameters of primer used for qPCR.

Name	Sequence	GC content (%)	Annealing temperature (°C)
*KLF9* forward	5′-TGGCTGTGGGAAAGTCTATGG-3′	52.4	60
*KLF9* reverse	5′-CTCGTCTGAGCGGGAGAACT-3′	60	60
*TPD52* forward	5′-GCTGCTTTTTCGTCTGTTGGCT-3′	50	60
*TPD52* reverse	3′-TCAAATGATTTAAAAGTTGGGGAGTT	30	60
miR223 forward	5′-AGCCGTGTCAGTTTGTCAAAT-3′	42.9	60
miR-223 reverse	5′-GTGCAGGGTCCGAGG TC-3′	70.6	60
*PKCϵ* forward	5′-AGCCTCGTTCACGGTTCT-3′	55.6	60
*PKCϵ* reverse	5′-TGTCCAGCCATCATCTCG-3′	55.6	60

### Statistical Analysis

Statistical analysis was performed with one-way and two-way ANOVA in order to show the relationship of the expressions of *TPD52*, *KLF9*, miR-223, and *PKCϵ* with the different clinicopathological features of cervical cancer. Spearman’s rho correlation was used to test the association of age and the stage of the disease. All these statistical tests were performed using GraphPad Prism 6.0 software. Similarly, GraphPad prism was employed for generating the receiver operating characteristic (ROC) curve.

### Kruppel-Like Factor 9: Three-Dimensional Structure Prediction

The 3D structure of KLF9 protein (NP_001197.1) was determined through homology modeling *via* SWISS-MODEL, a bioinformatics web server. For prediction of the 3D structure of KLF9, the first amino acid sequence of the *KLF9* gene was retrieved from the National Center for Biotechnology Information (NCBI) in FASTA format. In order to find the conserved domains and the evolutionary relationships between all the 17 members of the KLF family, multiple sequence alignment was done using Clustal Omega. For a better understanding of the evolutionary histories and conservation of the different members of the KLF family, phylogenetic analysis was performed using MEGA 7. The secondary structure of KLF9 was predicted *via* different servers, i.e., UCL Bioinformatics Group (34), SPIDER2 (35), and Predict Protein (36). For 3D structure predictions, *KLF4* was chosen as a template due to the fact that its structure has already been crystallographically predicted in RCSB-PDB (Research Collaboratory for Structural Bioinformatics Protein Data Bank). Hence, the structure of *KLF4* (PDB ID: 2BWU) was taken from RCSB-PDB. After acquistion of the template (*KLF4*) structure, the sequence of *KLF9* in FASTA format was aligned to the crystallographically determined structure of *KLF4 via* the SWISS-MODEL and a 3D model of *KLF9* was generated.

### Pathway Construction

In order to construct a genetic pathway depicting the crosstalk between understudied genes, the Kyoto Encyclopedia of Genes and Genomes (KEGG) database was used and STRING analysis was performed to study the gene linkage, while the genetic pathway was obtained *via* DAVID software.

## Results

### Kruppel-Like Factor 9: Three-Dimensional Structure Prediction

#### Multiple Sequence Alignment

**Figure 1:**
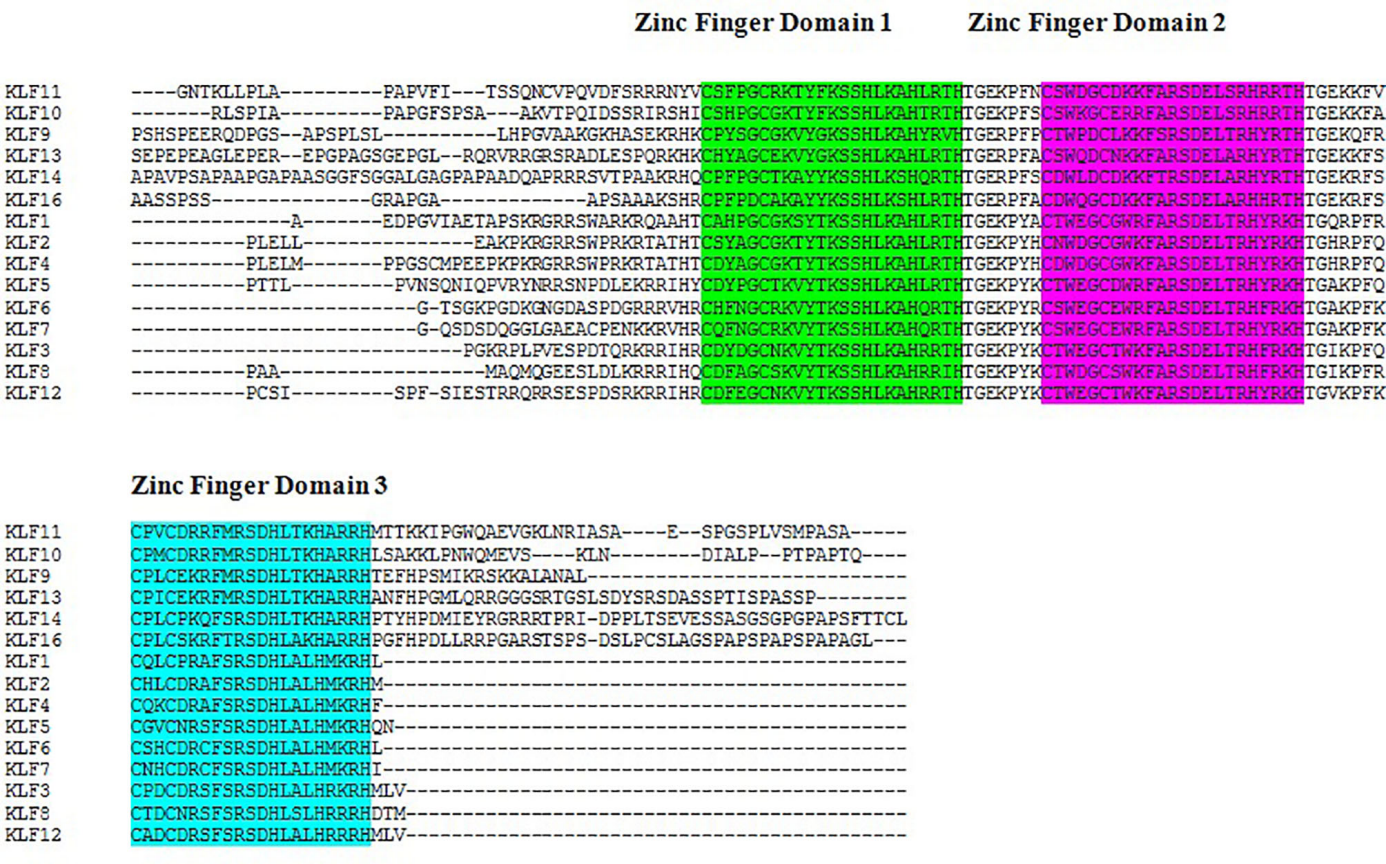
Sequence alignment of Kruppel-like factors depicting the conserved domains obtained from Clustal Omega. Certain sequence alignments have been deleted for formatting. Zinc figure domain 1 (labeled *green*), zinc figure domain 2 (labeled *purple*), and zinc figure domain 3 (labeled *blue*) are highlighted.

The results of the multiple sequence alignment of *KLF9* with the rest of the members of the KLF family *via* Clustal Omega (37) depicted the conserved domains across all KLF family members. **Figure 1** depicts the results of multiple sequence alignment using Clustal Omega. Three tandem C_2_H_2_ zinc finger domains, 1, 2, and 3, were found to be conserved throughout the members of the KLF family.

#### Phylogenetic Tree Construction

**Figure 2:**
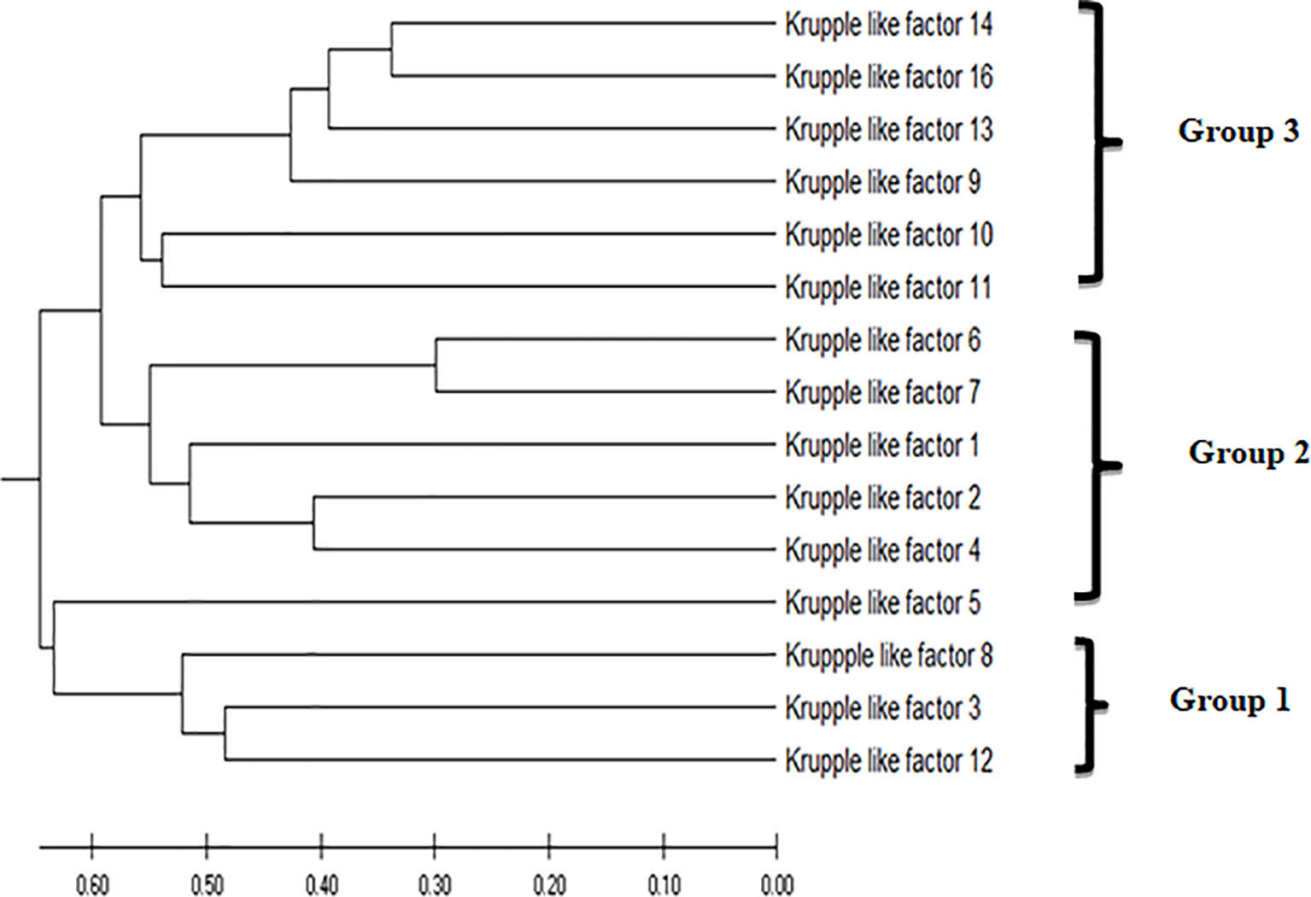
The evolutionary history was inferred using the unweighted pair group method with arithmetic mean (UPGMA). The optimal tree with the sum of branch length = 7.54632578 is shown. The tree is drawn to scale, with branch lengths in the same units as those of the evolutionary distances used to infer the phylogenetic tree. The evolutionary distances were computed using the Poisson correction method (39) and are in the units of the number of amino acid substitutions per site. This analysis involved 15 amino acid sequences. All ambiguous positions were removed for each sequence pair (pairwise deletion option). There were a total of 602 positions in the final dataset. Evolutionary analyses were conducted in MEGA X (40).

Phylogenetic analysis of the KLFs performed by MEGA 7 (38) using the UPGMA (unweighted pair group method with arithmetic mean) phylogenetic tree placed *KLF9* in group 3 based on its transcription repression activity (**Figure 2**). Like in earlier studies, the KLF family members were divided into three groups based on their evolutionary histories, structural characteristics, and binding domains, which help define their functions. Group 1 includes *KLF3*, *KLF8*, and *KLF12*. These members serve as repressors of transcription by mediating interactions with the co-repressors Sin3A and CtBP. Group 2 includes *KLF1*, *KLF2*, and *KLF4*–*KLF7*. These members act as activators of transcription. Group 3 includes *KLF9*–*KLF11*, *KLF13*, *KLF14*, and *KLF16*. These members serve as repressors of transcription by mediating interactions with the co-repressors Sin3A and CtBP (41).

#### Functional Binding Domains

Each member of KLF family, despite having highly conserved consensus sequences at the C-terminal region, has unique functions involved in cellular processes. This is due to great variations in sequences at the N-terminus region of KLFs that mediate interactions with diverse activators and repressors of transcription. The KLF sequences contain conserved motifs, at the N-terminus, comprising CtBP and Sin3A binding sites (41). Co-repressor C-terminal binding protein (CtBP) is a co-repressor of transcription. The main mechanism by which CtBP proteins suppress transcription is by recruiting histone methyl transferases and histone deacetylases (HDACs) to transcriptional complexes, which causes chromatin compaction and transcriptional silencing by the methylation and deacetylation of proteins, respectively (42, 43). *KLF3*, *KLF5*, *KLF8*, and *KLF12* contain the conserved motif CtBP binding site. *KLF3*, *KLF8*, and *KLF12* contain the conserved sequence PXDLS that mediates the interaction between KLFs and CtBP. This interaction facilitates the functions of *KLF3* and *KLF8* in co-repression and the activity of *KLF12* in repressing *AP-2α* gene expression (44). Sin3A is a protein that functions as a repressor of transcription. It is involved in the recruitment and binding of HDACs (45). *KLF9*, *KLF10*, *KLF11*, *KLF13*, *KLF14*, and *KLF16* possess binding sites for Sin3A. These KLFs possess the R1 domain that enclose a Sin3-interacting domain (SID), an α-helical hydrophobic structure that meditates binding with the PAH domain of Sin3 proteins (46). It was found that *KLF9*, *KLF10*, *KLF11*, *KLF13*, *KLF14*, and *KLF16* possess a conserved α-helical motif in their structure, i.e., AA/VXXL, a binding site for Sin3A that facilitates interaction with Sin3A and causes transcriptional repression (47). Unexpectedly, *KLF1* possesses no SID, but still interacts with Sin3A and acts as a co-repressor (48).

#### Sin3A Binding Site in *KLF9*

**Figure 3:**
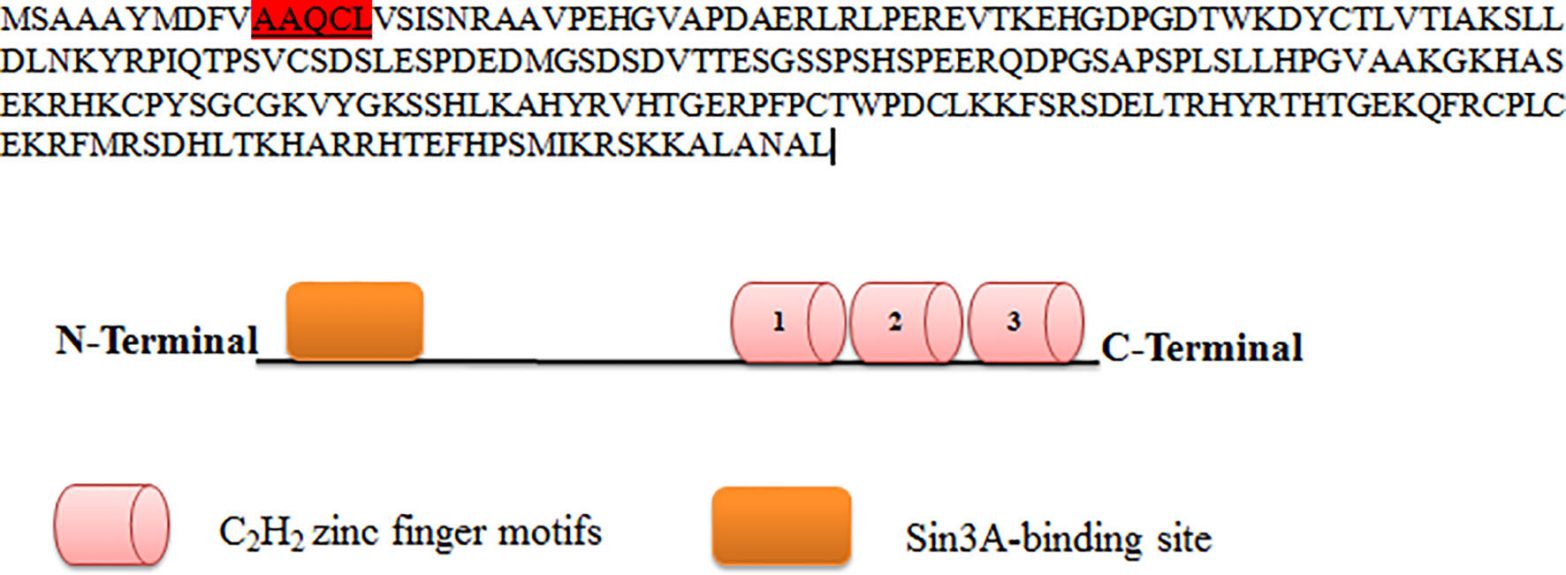
Protein sequence of *KLF9*. *KLF9* is highly homologous to other members of the Kruppel-like factor (KLF) family at carboxy-terminal DNA-binding regions, which contain three C_2_H_2_ zinc finger motifs. At the N-terminal region is the Sin3A binding region.

*KLF9* contains the conserved hydrophobic motif AAQCL in its amino acid sequence, as shown in **Figure 3**. It serves as a SID and is able to recruit and bind Sin3A. Sin3A proteins bind HDAC1, HDAC2, and other proteins, probably assembling multi-unit complexes (HDAC1 and HDAC2), altering chromatin compaction and so repressing transcription. A number of studies have justified the presence of such conserved motifs in *KLF*9 (49).

#### Subcellular Localization

**Figure 4:**
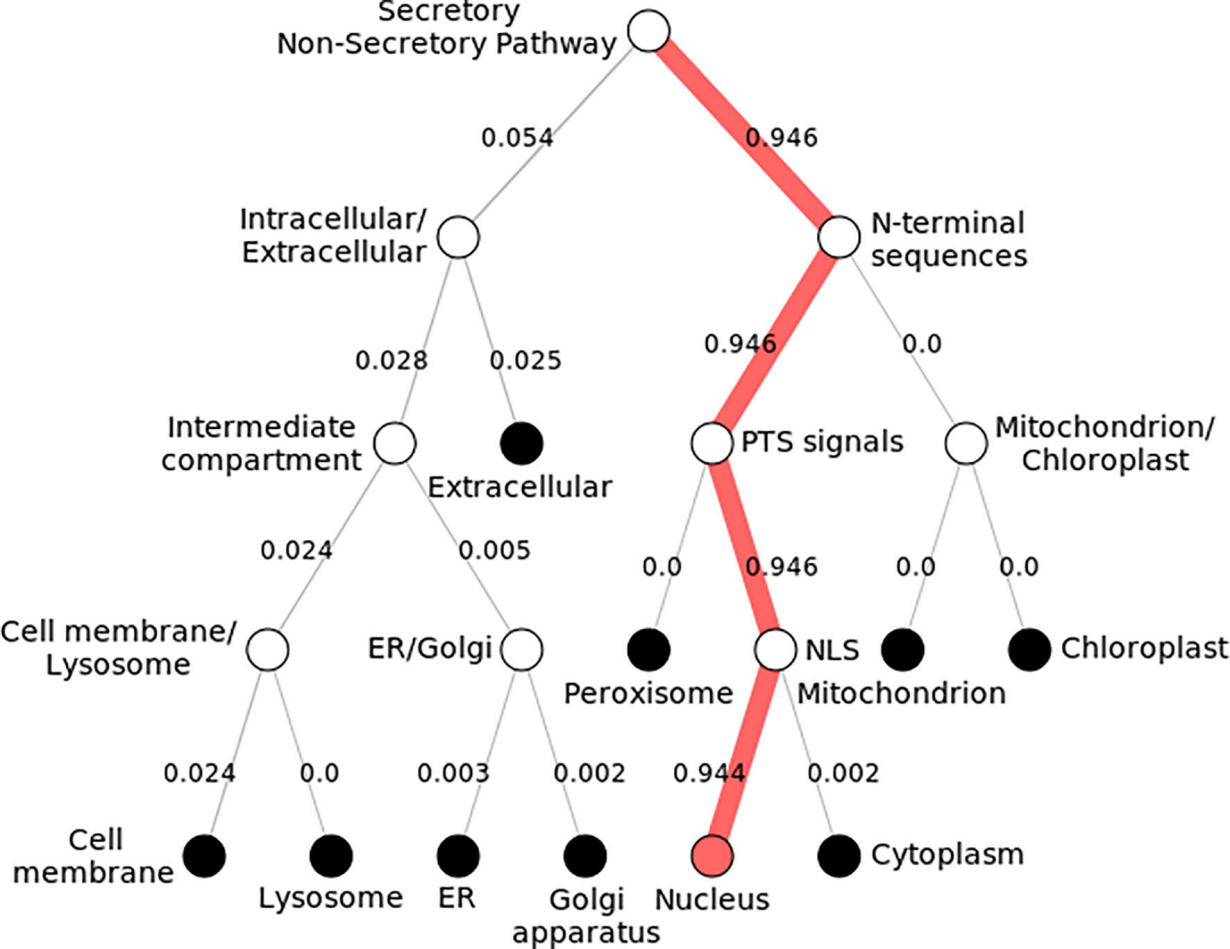
Subcellular localization of KLF9. Pathway following subcellular localization of KLF9 generated by DeepLoc-1.0. Numerous locations are shown, and each follows a distinct pathway and score. The KLF9 protein is localized inside the nucleus (depicted by 0.9 score). It directs toward the nucleus by executing peroxisomal targeting signals (PTS) and nuclear localization signals (NLS).

Subcellular localization of *KLF9* was found to be inside the nucleus (**Figure 4**). By modeling the functional domain features and the hidden associations of gene ontology, different servers gave different nuclear signals. Hum-mPLoc 3.0 showed a nuclear signal of 1.88, while DeepLoc-1.0 showed a nuclear signal of 0.99.

#### 3D Structure Visualization and Assessment of KLF9 Protein

**Figure 5:**
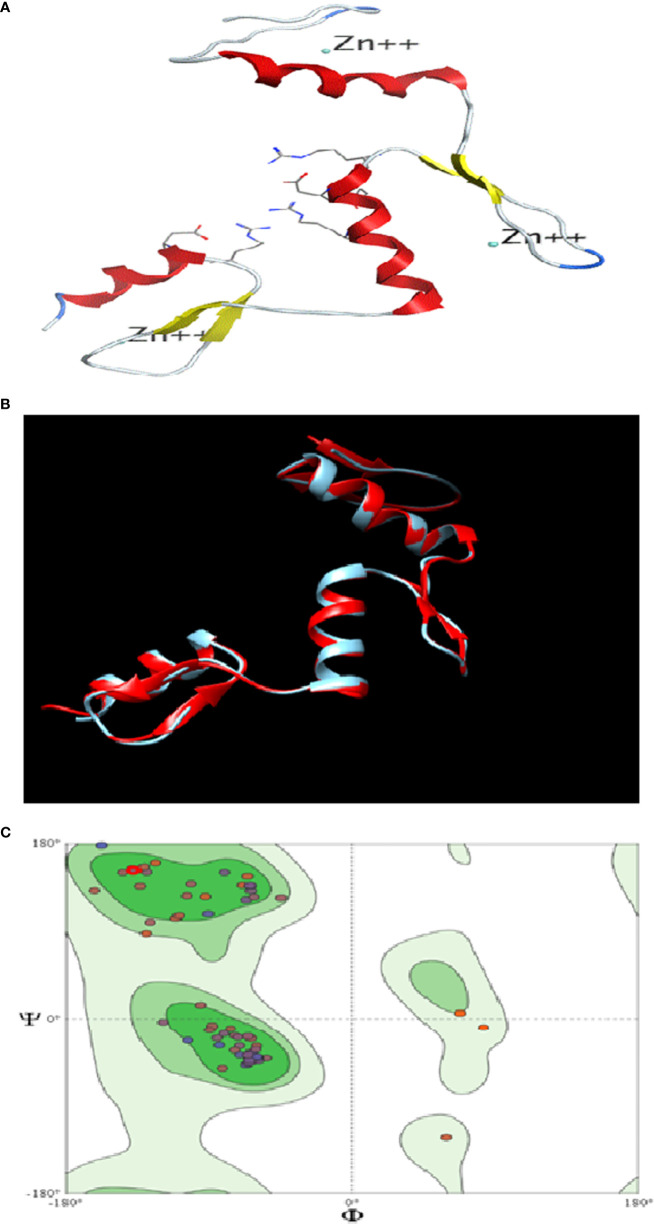
*In silico* analysis of *KLF9*. **(A)** Three-dimensional structure of *KLF9*. **(B)** Comparison of the crystallographically determined structure 2bwu (labeled *red*) and the predicted structure *KLF9* (labeled *blue*) for the analysis of structure conservation. **(C)** Ramachandran plot analysis determining the quality of the model. Most amino acids (95%) were found in favored regions, showing that the model is of good steriochemical quality.

The similarity index between the structures of the template (KLF4) and target (KLF9) was found to be 62%. The 3D structure of KLF9 is shown in **Figure 5A**. Using Chimera, the structure of KLF9 obtained *via* the SWISS-MODEL was superimposed on KLF4 (template) for the anlysis of structural conservation between the target (KLF9) and template (KLF4). The template is labeled red, while target is labeled blue. **Figure 5B** illustrates the superimposed structures of the template (KLF4) and target (KLF9) proteins. Ramachandran plots were used to analyze the quality of the model obtained *via* the SWISS-MODEL. These plots were used for visualization of the dihedral angles, i.e., phi (*φ*) and psi (*ψ*) angles of the amino acids. It was found that most of the amino acids were found to be lying in favorable regions, i.e., 95.06%, and Ramachadran outliers were 1.23% (A146 PRO). Bad bonds in the structure were 0/721, while bad angles were 16/965. **Figure 5C** illutrates the Ramachadran plot.

### Expressions of *TPD52, KLF9*, miR-223, and *PKCϵ* in Blood of Cervical Cancer Patients

**Figure 6:**
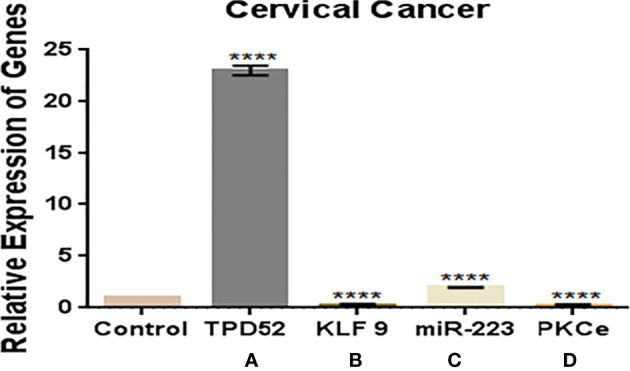
Expressions of *TPD52*, *KLF9*, miR-223, and *PKCϵ* in blood of cervical cancer patients. **(A)** *TPD52* expression was increased 23-fold. **(B)** *KLF9* expression was decreased 0.4-fold. **(C)** miR-223 expression was increased 2-fold. **(D)** *PKCϵ* decreased 0.05-fold. Fold change is plotted on the *y*-axis and study groups on the *x*-axis. Illustrative data are presented as the mean ± SEM of triplicate experimentations. Statistical significance was measured by ordinary two-way ANOVA (****p < 0.0001).

In this study, we observed a significantly increased expression of *TPD52* (23.8 ± 0.42) in understudied samples of cervical cancer compared to the controls. The expression of *KLF9* was found to be downregulated in the blood of cervical cancer patients (0.14 ± 1.6) relative to healthy controls. There was an elevation of miR-223 expression in cervical cancer patients (2.0 ± 1.8) relative to controls. In the case of *PKCϵ*, its expression was found to be significantly reduced in cervical cancer patients (0.05 ± 5.7). Overall, we found that the expressions of *TPD52* and miR-223 were increased 23- and 2-fold in peripheral blood of cervical cancer patients, respectively, whereas expressions of *KLF9* and *PKCϵ* were 0.14- and 0.05-fold reduced in cervical cancer patients relative to healthy individuals (**Figure 6**).

### Relative Expressions of TPD52, KLF9, miR-223, and PKCϵ With Clinical Features in Cervical Cancer

**Figure 7:**
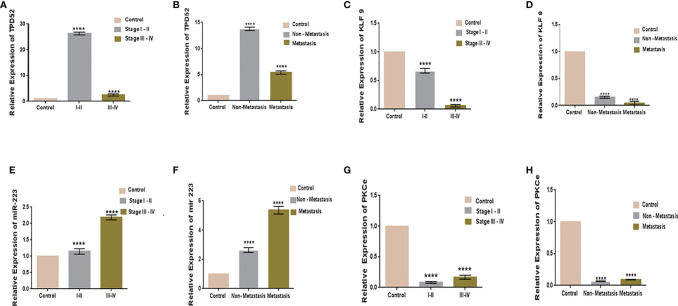
Relative gene expression with clinical features of cervical cancer. Relative *TPD52* expression with tumor stage **(A)** and metastasis **(B)**. Relative *KLF9* expression with tumor stage **(C)** and metastasis **(D)**. Relative miR-223 expression with tumor stage **(E)** and metastasis **(F)**. Relative *PKCϵ* expression with tumor stage **(G)** and metastasis **(H)**. Fold change is plotted on the *y*-axis and study groups on the *x*-axis. Illustrative data are presented as the mean ± SEM of triplicate experimentations. Statistical significance was measured by ordinary one-way ANOVA (****p < 0.0001).

The clinicopathological features of cervical cancer patients are shown in **Table 2**. The relative expressions of *TPD52*, *KLF9*, miR-223, and *PKCϵ* in cervical cancer patients were measured with respect to their clinical features. The fold change and expression status of *TPD52*, *KLF9*, miR-223, and *PKCϵ* for each clinicopathological feature, i.e., low tumor stage groups I–II and advanced tumor stage groups III–IV, distant metastatic *vs*. non-metastatic group, and treatment status of patients (e.g., chemotherapy, radiotherapy, or chemoradiotherapy), are shown in **Table 3**. Significant results (*p* < 0.001) were found between all groups of patients. The expression of *TPD52* was found to be significantly higher in the lower tumor stage and non-metastatic groups of patients in comparison to its high expression in the advanced tumor stage and distant metastatic groups of patients (**Figures 7A, B** **)**. A similar trend was found for miR-223 (**Figures 7E, F** **)**. In the case of *KLF9*, its expression was much more significantly reduced in the advanced tumor stage and distant metastatic groups relative to its less reduced expression in the lower tumor stage and non-metastatic groups (**Figures 7C, D** **)**. On the other hand, for *PKCϵ*, its expression was much more significantly reduced in the lower tumor stage and non-metastatic groups relative to its less reduced expression in the advanced tumor stage and distant metastatic groups (**Figures 7G, H** **)**.

**Table 2:** Clinicopathological features of the cancer patients enrolled in the study.

Clinicopathological characteristics		Cervical cancer
		*N* (%)
Age (years)	≤50	52 (52)
	>50	48 (48)
Stage	I–II	48 (48)
	III–IV	52 (52)
Metastasis	Metastatic	38 (38)
	Non-metastatic	62 (62)
Treatment	Chemotherapy	16 (16)
	Radiotherapy	32 (32)
	Chemotherapy + radiotherapy	52 (52)

**Table 3:** Relationship between *TPD52*, *KLF9*, *PKCϵ*, and miR-223 expression and clinicopathological features of cervical cancer.

Clinical–pathological characteristics of cervical cancer patients			*TPD52* expression			*KLF9* expression			miR-223 expression			*PKCϵ* expression		
Features	Groups	*N* (%)	Expression status	Fold change	*p*-value	Expression status	Fold change	*p*-value	Expression status	Fold change	*p*-value	Expression status	Fold change	*p*-value
**Stage**	I–II	48 (48)	High	27.0614	0.0001	High	0.68388	0.0001	High	1.2246	0.0001	High	0.05228	0.0001
	III–IV	52 (52)	Low	1.62668	0.0001	Low	0.01752	0.0001	Low	2	0.0001	Low	0.10324	0.0001
**Metastasis**	Metastatic	40 (40)	High	5.25275	0.0001	High	0.00733	0.0001	High	5	0.0001	High	0.08387	0.0001
	Non-metastatic	60 (60)	Low	14.2051	0.0001	Low	0.13664	0.0001	Low	2.7869	0.0001	Low	0.07114	0.0001

**Figure 8:**
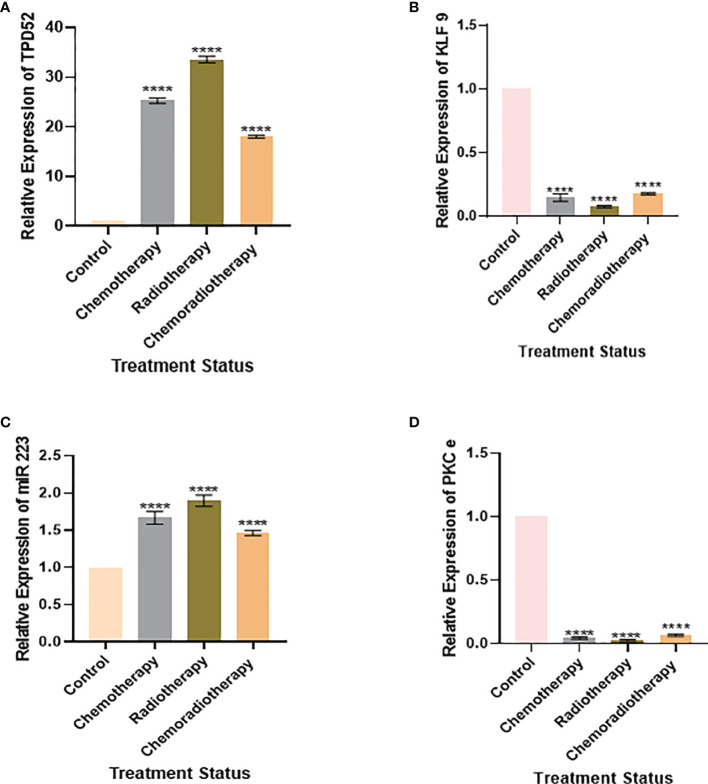
Relative gene expression with treatment status. **(A)** Relative *TPD52* expression. **(B)** Relative *KLF9* expression. **(C)** Relative miR-223 expression. **(D)** Relative *PKCϵ* expression. Illustrative data are presented as the mean ± SEM of triplicate experimentations. Fold change is plotted on the *y*-axis and study groups on the *x*-axis. Statistical significance was measured by ordinary one-way ANOVA (****p < 0.0001).

We also found that the expression of *TPD52* was lowest in patients undergoing chemoradiotherapy relative to its higher expression in patients receiving a combination of chemotherapy and radiotherapy (**Figure 8A**). A similar trend was followed in the expression profile of miR-223 (**Figure 8C**), whereas for *KLF9* and *PKCϵ*, patients undergoing chemoradiotherapy showed higher expressions relative to patients on chemotherapy and radiotherapy, where their expressions were significantly reduced (**Figures 8B, D** **)**. However, it is to be noted that the expressions of *TPD52* and miR-223 were higher relative to healthy controls and that the expressions of *KLF9* and *PKCϵ* were lower in comparison to healthy controls in each group of patients.

### Specificity of *TPD52*, *KLF9*, miR-223, and *PKCϵ* for Cervical Cancer Diagnosis

**Figure 9:**
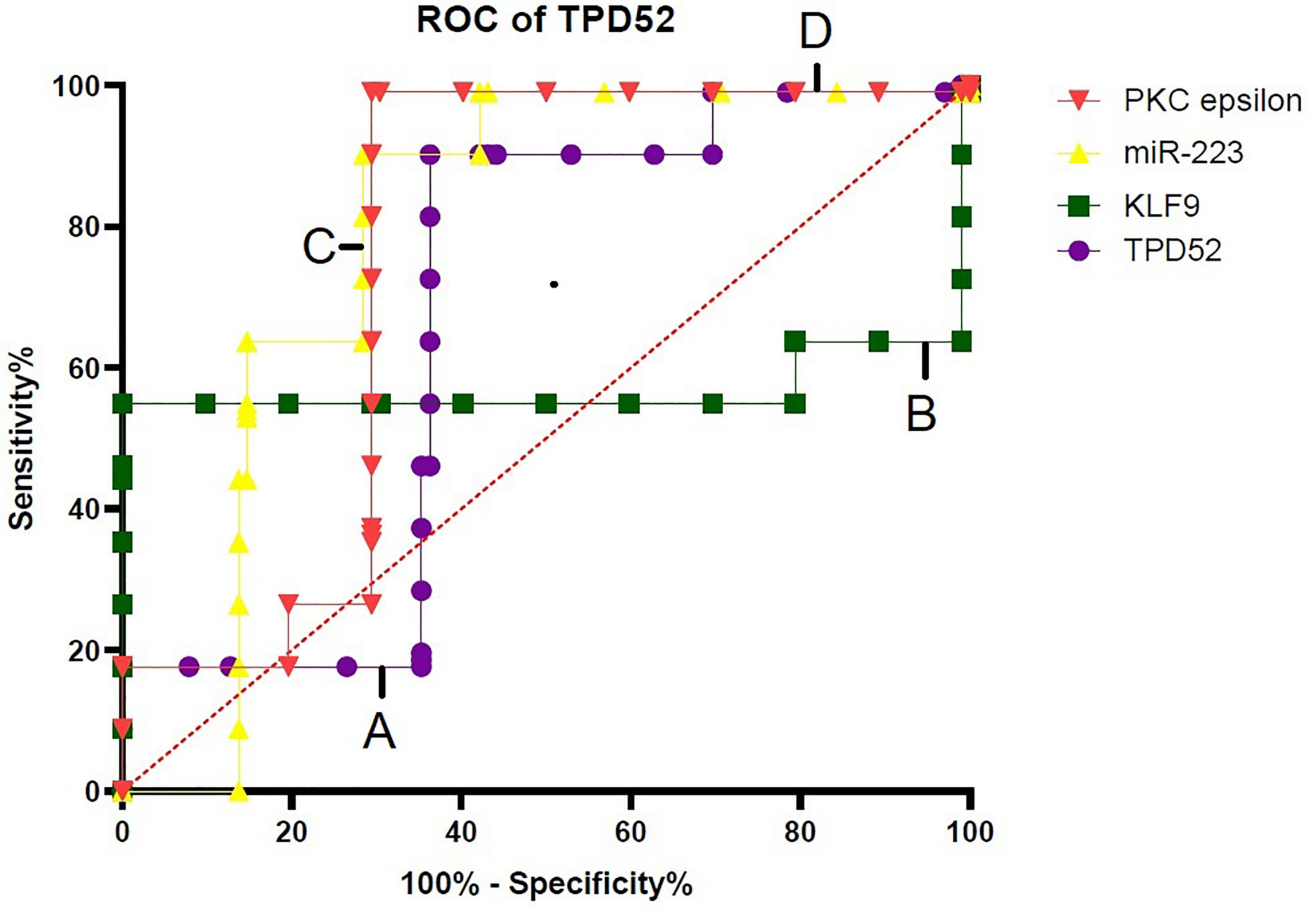
Specificity of *TPD52*, *KLF9*, miR-223, and *PKCϵ* in the diagnosis of cervical cancer. Receiver operating characteristic (ROC) curve for *TPD52*, *KLF9*, miR-223, and *PKCe* predicted high risk of cervical cancer. **(A)** *TPD52*: area under the ROC curve (AUC) = 0.6685 and 95% confidence interval (CI) = 0.5880–0.7490. **(B)** *KLF9*: AUC = 0.5706, 95%CI = 0.4775–0.6638. **(C)** miR-223: AUC = 0.7884, 95%CI = 0.7184–0.8583. **(D)** *PKCϵ*: AUC = 0.7595, 95%CI = 0.6852–0.8338.

For verification of the relationship between these blood-based biomarkers (*TPD52*, *KLF9*, miR-223, and *PKCϵ*) and cervical cancer, ROC curves were generated (**Figure 9**). The area under the ROC curve (AUC) was calculated and 95% confidence intervals were determined.

### Association Between Patient Age and Cancer Stage

**Figure 10:**
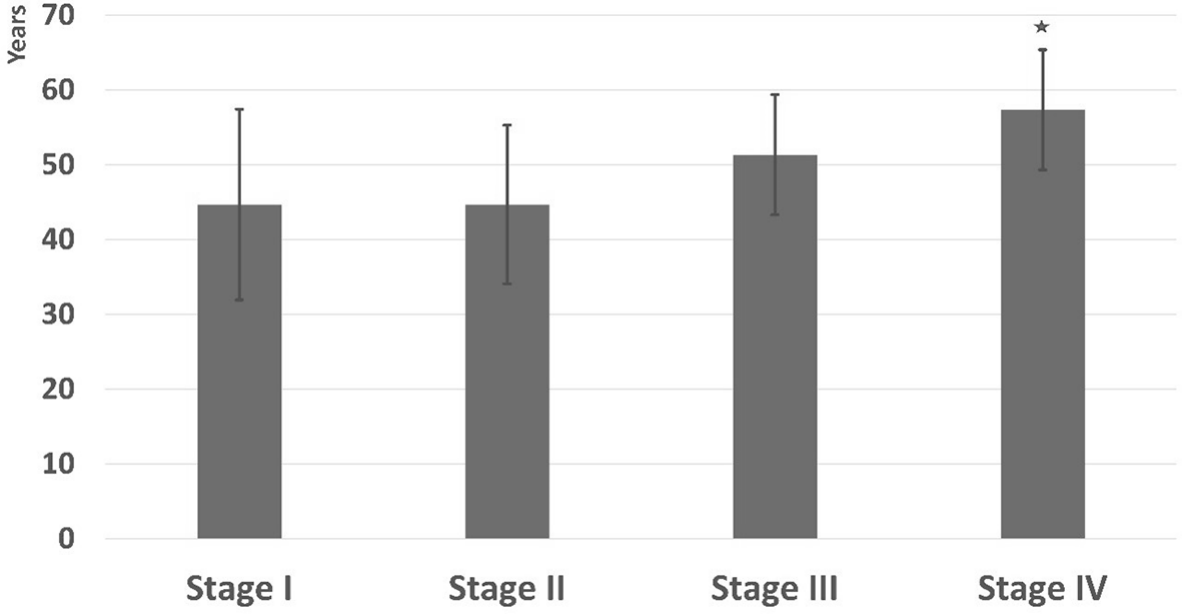
Age of participants in different clinical stages. *Significant *vs*. stage I.

The association between patient’s age and cancer stage is shown in **Figure 10**. Participants diagnosed with stage IV were significant older than those in the early stages. Furthermore, age showed a significant positive correlation with stage (*r* = 0.503, *p* < 0.001).

## Discussion

Tumors arising in the genital tract of females were found to be the fourth most frequent set of malignancies among females. The absence of screening methods, diagnostic techniques, and treatment and the lack of proper knowledge are the leading causes of cervical cancer incidences (50). The late diagnosis of the illness results in increased mortality rates (51). Although various screening techniques are being used for the diagnosis of cervical cancer, the death rates in developing states continue to be high, i.e., 87%. Pap smear is currently used for screening cervical neoplasia at an early stage. However, the false-negative results that are often produced by the Pap test is one of its major drawbacks (50). Hence, discovering the biological and molecular mechanisms of tumor progression and identifying diagnostic biomarkers have become essential in cancer research studies.

With improvements in technology, there has been a significant increase in the structure determination of numerous proteins. Still, the prediction of protein structures remains a challenging task. However, certain theoretical models can be used to assess the topological characteristics of proteins. The 3D structure of protein helps in understanding their functions and their interactions with their binding partners. Homology modeling can help in predicting low-resolution structures. Hence, in this study, the 3D structure of *KLF9* was predicted *via* the SWISS-MODEL Workspace. The template used for 3D structure predictions was *KLF4*. The server used for the visualization of the 3D structures was Chimera. The similarity index between the structure of a template (*KLF4*) and a target (*KLF9*) was known to be 62%, and no bad bonds were found in the predicted structure. This study also predicted the possible crosstalk of *KLF9* with *TPD52*, miR-223, and *PKCϵ*. KEGG and STRING were used to determine gene linkage with neighboring genes, while DAVID 6.8 was used to dig out the biological meaning from a large set of genes.

**Figure 11:**
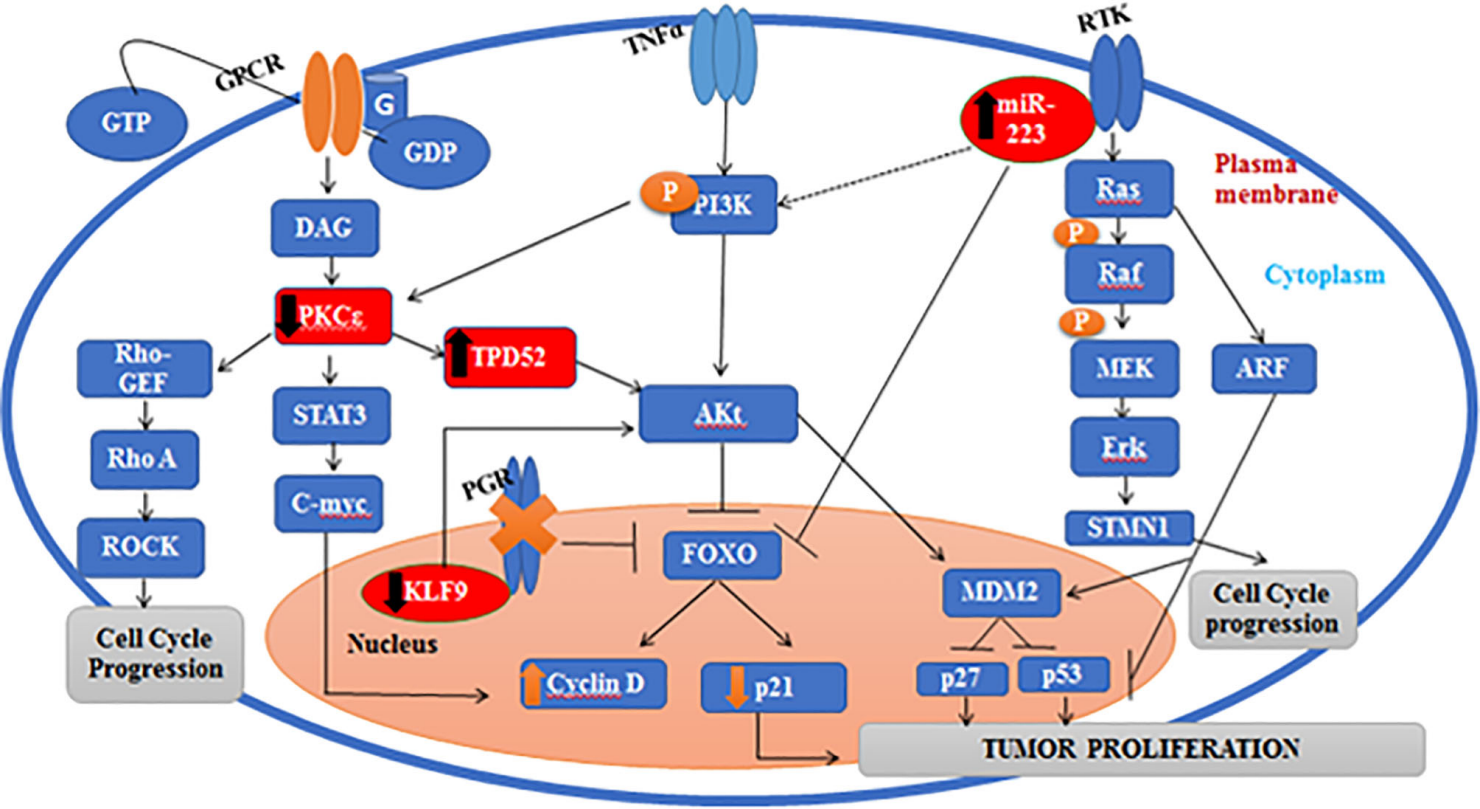
Pathway showing the correlations between *TPD52*, *KLF9*, miR-223, and *PKCϵ*.

Gene linkage analysis *via* KEGG and STRING is shown in **Figure 11**. Our genetic pathway depicted that all the understudied genes are linked to each other and are involved in the Akt pathway. The pathway obtained *via* DAVID software depicted that *PKCϵ* is found is upstream to the Ras/Raf pathway and bridges the activation of this pathway by GPCRs. Some studies have also described the involvement of *PKCϵ* in the Ras/Raf pathway and have revealed that *PKCϵ* activates GPCR coupled Ras/Raf pathway and helps in the remodeling of cardiomyocytes (24). We also found that the regulation of *PKCϵ* by the *STAT3* gene (signal transducer and activator of transcription 3) stimulates the activity of cyclin D in the nucleus *via* activation of o-myc (family of transcription regulatory genes), which leads to enhanced cell cycle progression. A regulatory link of *PKCϵ* with *STAT3* has also been established in prostate adenocarcinoma (52). A few studies also depicted the activation of *STAT3 via* Rho kinases, which validates our results (53). Moreover, *TPD52* also activates *STAT3*. A recent study has ascertained the activation of *STAT3 via TPD52* (16). Hence, the transcriptional activity of *STAT3* is regulated by *PKCϵ*, *TPD52*, and Rho-kinases. *PKCϵ* involvement was also found in the Rho signaling pathway, which eventually leads to metastasis. According to a recent study, *PKCϵ* also facilitates metastasis in breast cancer by activating Rho-GTPases (54). Our genetic pathway showed that Rho-GTPases are found downstream of *PKCϵ*, and ERK phosphorylation in the Ras/Raf pathway occurs due to the activation of a downstream target of PKCϵ (Rho GTPases). Our finding is in agreement with the previously published report by Pan et al. (55), who also found the same phosphorylation mechanism of ERK in the Ras/Raf pathway. Our genetic pathway also depicted the involvement of *PKCϵ* in the Akt pathway. We found that *PKCϵ* is located upstream of *TPD52*, and both of these genes activate the Akt pathway, which promotes tumor proliferation and invasion. The role of *PKCϵ* in Akt activation, by phosphorylating Akt at serine 473, has already been established (56). Akt is known to regulate proliferation and the cell cycle by targeting cyclin D1, p21, p53, and p27 (57). Forkhead box O (FOXO) is a transcription factor that serves as a downstream target of Akt (protein kinase B). Akt inhibits FOXO by phosphorylating it, and hence promoting cell survival, growth, and proliferation. *TPD52* and *PKCϵ* block the transcriptional activity of FOXO, activate cyclin D, and inactivate p27 (regulator of the cell cycle), leading to enhanced cellular proliferation. According to Zhang et al. (58), the PI3K/Akt signaling pathway inactivates FOXO and, hence, cause the downregulation of cell cycle controls, i.e., CDKI and p27. Our results manifested that the decreased expression of *KLF9* inhibits the progesterone growth hormones (progesterone receptor gene, PGR), which in return directly blocks FOXO and ultimately promotes tumorigenesis. Pabona et al. (59) validates our finding by demonstrating *KLF9* as a regulator of PGR. Loss of *KLF9* leads to the inhibition of PGR and FOXO signaling, hence leading to oncogenesis and tumor invasion in endometrial cells. The genetic pathway constructed in the current study also proposes that the increased expression of miR-223 causes the activation of STMN1 and inhibition of FOXO. In gastric cancer, overexpression of miR-223 also leads to a reduced expression of FOXO and the inhibition of cyclin D, p21, and p27 (60). Moreover, miR-223 is also involved in the activation of phosphatidylinositol 3-kinase (PI3K), which in return produces phosphatidylinositol triphosphate (PIP3) in the cell membrane. PIP3 activates Akt signaling. Zhu et al. (8) also reported on the role of overexpressed miR-223 in the activation of Akt and onset of tumorigenesis in cervical cancer.

This study also aimed to identify new biomarkers and critical genes linked to the prognosis and diagnosis of cervical cancer. In our study, we have measured the co-expressions of *TPD52*, *KLF9*, miR-223, and *PKCϵ* in cervical cancer. Expression dysregulation of the biomarkers *PKCϵ*, *TPD52*, miR-223, and *KLF9* was determined by comparing the expression fold change with the expression profile of the healthy group. Previously, numerous studies that determine the expressions of biomarkers in patient blood using real-time-PCR were conducted. For instance, the prognostic significance of *KLF7* was studied in tongue cancer (61). The plasma levels of several miRNAs, such as miR-218, miR-223, miR-7, miR30, and miR-21, were studied in hepatocellular carcinoma and gastric and ovarian cancer (62–64). Recently, the relative expressions of matrix metalloproteinases (MMPs) in blood of breast cancer patients were investigated to determine their role in cancer progression (65), hence indicating their possible application in disease prognosis. The current study also evaluated the mRNA expression of these molecules in blood of cervical cancer patients and provided a foundation for conducting an in-depth, proteome-level analysis *in vitro* and *in vivo*. The outcome of the current study indicated the prognostic significance of these molecules for cervical cancer. The diagnostic specificity of these biomarkers was also determined through ROC curve analysis. However, further evaluation on a larger cohort size and at the protein level is required to determine its clinical significance.

Earlier, the role of understudied genes had been independently studied in various tumors, which confirmed the involvement of these genes in cancer, metastasis, and expansion and in resistance to therapy. To the best of our knowledge, the co-expression of these genes in cervical cancer has not been studied yet. We observed an increased expression of *TPD52* in cervical cancer patients relative to healthy controls who have very low levels of the *TPD52* gene in their blood. Various studies reported the upregulation of *TPD52* expression in quite a few cancers, such as breast, prostate, and pancreatic cancer, Burkitt’s lymphoma, multiple myeloma, and melanoma (12). On the other hand, the expression of *TPD52* is downregulated in some cancers, such as papillary renal cell cancer, lung cancer, and liposarcoma (13). In the case of *KLF9*, we observed its significantly reduced expression in cervical cancer patients relative to healthy controls. Similar downregulation of *KLF9* has been reported in endometrium cancer, where its downregulation is linked to estrogen-mediated growth control (66). The reduced expression of *KLF9* has also been reported in breast cancer, human colorectal tumors, and hepatocellular carcinoma (67). Various studies have discovered that expression profiling of various circulating miRNAs in the blood may probably be used in therapeutic interventions and in identifying different tumor types (68). We have found an upregulation of miR-223 in cervical cancer patients relative to the healthy individuals. According to a recent study, the expression of miR-223 is significantly elevated in gastric adenocarcinoma cells. The upregulation of miR-223 encouraged cell proliferation and reduced apoptosis in gastric adenocarcinoma cells, while the downregulation of miR-223 expression has been linked to various cancer subtypes, including leukemia and gastric, esophageal, and colorectal cancer (69). In the case of *PKCϵ*, we observed its reduced expression in cervical cancer patients relative to healthy controls who had significantly high levels of this gene in their blood. On the contrary, an upregulation of *PKCϵ* has been reported in a large number of carcinomas, including breast, lung, and prostate cancer (70). Various reports have confirmed the role of this gene as an oncogene and its involvement in tumor metastasis (55). Our study found that the expression of *TPD52* was upregulated in the advanced-stage tumor group (1.62 ± 0.4) and in the distant metastatic group of patients (5.25 ± 0.42) relative to lower stage tumor and non-metastatic groups, where its expression levels were increased 27.0 ± 1.68- and 14.2 ± 1.68-fold, respectively. Hence, *TPD52* may serve as a potent early diagnostic biomarker in cervical cancer. A recent study has reported the decreased expression of *TPD52* in tumorous tissues of hepatic cellular carcinoma (HCC) in comparison to healthy tissues. Further correlation analysis exposed that the reduced expression of *TPD52* in HCC was suggestively linked to advanced stage tumor, signifying that a reduced *TPD52* expression may promote tumor metastasis (71). These results are inconsistent with our study. Furthermore, in the case of *KLF9*, we observed its reduced expression in advanced tumor stage (0.01 ± 1.6) and in distant metastasis (0.007 ± 1.39). A downregulated expression of *KLF9* was suggestively found in the lower stage tumor group (0.68 ± 1.6) and the non-metastatic group (0.13 ± 1.82). Our result is encouragingly inconsistent with recent findings that point to the fact that a reduced expression of *KLF9* is linked to poor survival and prognosis in pancreatic ductal adenocarcinoma and leads to tumor metastasis (9). Our study found that the expression of miR-223 was increased in the advanced tumor stage (2.07 ± 3.9) and distant metastasis (5.8 ± 4.25) groups, while its expression was decreased in the lower tumor stage group (1.2 ± 43.9) and the non-metastatic group (2.7 ± 4.5). Further studies have revealed that miR-223 plays a significant part in the metastasis of cervical cancer. The upregulation of miR-223 promotes metastasis in cervical cancer cells (72). These results validate our results showing that the increased expression of miR-223 in cervical cancer patients causes metastasis and poor prognosis. The expression of *PKCϵ* was much more downregulated in the advanced tumor stage (0.10 ± 5.8) and distant metastasis (0.08 ± 6.36) groups relative to the lower tumor stage group and the non-metastatic group, where its expression was reduced 0.05 ± 6.0- and 0.07 ± 5.87-fold, respectively. According to recent studies, *PKCϵ* causes tumor metastasis to the bone by promoting translation increase and causes osteosarcoma metastasis (73). These findings contradict our study as *PKCϵ* inhibited metastasis in cervical cancer. The contradictory results may be due to the different cancer types.

Our study also discovered the effect of treatment on the expression profiles of understudied genes. It was found that patients undergoing chemoradiotherapy showed better prognosis. In the case of *TPD52*, patients undergoing chemoradiotherapy showed the lowest expression (18.52 ± 1.84) relative to patients on chemotherapy (26.2 ± 1.5) and radiotherapy (34.7 ± 1.83). Likewise, patients on chemoradiotherapy showed the lowest miR-223 expression (1.51 ± 3.8) relative to patients undergoing chemotherapy (1.76 ± 3.7) and radiotherapy (2.03 ± 4.2). These results show patients’ response to treatment and indicate that chemoradiotherapy has better prognosis, while radiotherapy is linked to poor prognosis in cervical cancer. During treatment expression profiling, *KLF9* and *PKCϵ* were found to be slightly less reduced in patients treated with chemoradiotherapy, who showed better prognosis, relative to chemotherapy and radiotherapy. The expression patterns of *KLF9* in patients undergoing chemotherapy and radiotherapy were found to be 0.10 ± 0.60 and 0.08 ± 1.85, respectively. In the case of *PKCϵ*, these were found to be 0.06 ± 0.1 and 0.04 ± 6.48, respectively. Hence, it was deduced that chemoradiotherapy is linked to better survival of cervical cancer patients.

To further validate our findings, Spearman’s rho correlation was used to test the association of age and the stage of the disease. The association of age and stage of the disease was found in line with the frequency found in the literature in adults (74) and children (75). However, some studies showing evidence of a relationship between age and cancer in adults (76) have reported that cancer does not have to be a consequence of old age.

All the involved genes and miRNAs in our study are known to be implicated in various cancer signaling pathways, such as the PI3K/Akt, nuclear factor-κB, Wnt/β-catenin, and Ras signaling pathways. Hence, these genes and miRNAs may serve as potential diagnostic and prognostic biomarkers. Moreover, these genes can further be investigated as targets for anticancer therapy.

## Conclusion

In the present study, we identified the conserved domains and the 3D structure of *KLF9* and developed a genetic pathway establishing the crosstalk between *KLF9* and its upstream and downstream targets. Moreover, upregulation of the expressions of *TPD52* and miR-223 and downregulation of the expressions of *KLF9* and *PKCϵ* were found in peripheral blood of cervical cancer patients. Altered expressions of these genes have been found to be related to tumor progression. Alterations in the expression levels of the understudied genes in cervical cancer may serve as a potential circulating biomarker for cancer diagnosis and prognosis. Hence, understanding the functions, signaling pathways, and genetic networks of *TPD52, KLF9*, miR-223, and *PKCϵ* may synergistically reveal the mechanisms of disease progression and serve as a target for inhibitors, therefore assisting in the development of effective anticancer therapy.

## Data Availability Statement

The original contributions presented in the study are included in the article/supplementary materials. Further inquiries can be directed to the corresponding authors.

## Ethics Statement

The studies involving human participants were reviewed and approved. This experimental protocol for the use of human was approved (ref. no. IRB-110) by the Ethical Committee of Combined Military Hospital and ASAB, NUST. The patients/participants provided written informed consent to participate in this study. Written informed consent was obtained from the individual(s) for the publication of any potentially identifiable images or data included in this article.

## Author Contributions

MS, SS, KZ, YB, KK, NA and SR designed and conceived the study and analyzed the results. ED, SR, TA, MS, KK and AA conceived an initial part of the study, performed the experiment and histology, and helped in compiling the results. MS, KZ, and SS performed experiments. MS, SR, ED, AA, NA and TA helped in writing the results. SR, TA, DD, and AA wrote the paper with input from all other authors. MS, KZ, SR, YB, DD, SS, TA, KK, NA and AA made a substantial contribution in the interpretation of data and revised the manuscript for intellectual content. All authors read and approved the final manuscript.

## Funding

We are grateful to the Deanship of Scientific Research at King Saud University for funding this research through Research Group Project no. RGP-193. Furthermore, we are grateful to the Department of Healthcare Biotechnology, Atta-ur-Rahman School of Applied Biosciences, National University of Sciences and Technology, Islamabad, Capital, Pakistan, and Higher Education Commission for their funding through grant no. 10067. The funding body has no role in designing the study.

## Conflict of Interest

The authors declare that the research was conducted in the absence of any commercial or financial relationships that could be construed as a potential conflict of interest.

## Publisher’s Note

All claims expressed in this article are solely those of the authors and do not necessarily represent those of their affiliated organizations, or those of the publisher, the editors and the reviewers. Any product that may be evaluated in this article, or claim that may be made by its manufacturer, is not guaranteed or endorsed by the publisher.
